# Advice for lay callers with low-risk poison exposures by a regional poison control center: the impact on health care expenditures

**DOI:** 10.1186/s13690-022-00994-0

**Published:** 2022-11-30

**Authors:** Franziska Thal, Thomas Reinhold

**Affiliations:** 1 Kaufmännische Centrumsleitung CC05, Charité – Universitätsmedizin Berlin, Berlin, Germany; 2 Institut für Sozialmedizin, Epidemiologie und Gesundheitsökonomie, Charité – Universitätsmedizin Berlin, Luisenstraße 57, 10117 Berlin, Germany; 3 Institute for Complementary and Integrative Medicine, University Hospital Zurich and University of Zurich, Zurich, Switzerland

**Keywords:** PCC, Germany, Poisoning, Decision tree, Health care costs, Cost–benefit-analysis

## Abstract

**Background:**

Since establishing the first poison control centers (PCCs), there is a still ongoing debate regarding their relevance and financing. The present study aims to analyze whether a regional PCC can reduce the economic burden associated with utilization of health care structures due to low-risk poison exposures on the German health care system.

**Methods:**

A decision-tree based cost–benefit analysis comparing a situation utilizing PCC consultation versus a hypothetical situation without PCC consultation for low-risk poison exposures from the German health care system's perspective was conducted. The model inputs were obtained by a representative telephone survey of lay callers supplemented by empirical PCC and literature data. A probabilistic and deterministic sensitivity analysis with varying input variables was performed to prove the robustness of the findings.

**Results:**

In the underlying telephone survey, data of 378 lay callers could be considered and included in the decision tree model. As a result, the mean costs for handling one low-risk poison exposure case were €41.99 utilizing PCC consultation compared to €145.92 without PCC consultation, indicating a cost–benefit ratio of 3.48 for the existence of the PCC. The sensitivity analysis proved that the outcome of the decision analysis does not change significantly with varying inputs.

**Conclusion:**

The existence of PCCs relieve the burden on other health care providers and reduce health care costs to a relevant extent. Therefore, PCCs should be considered as an important supporting structure of the German health care system.

**Supplementary Information:**

The online version contains supplementary material available at 10.1186/s13690-022-00994-0.

## Introduction

The debate about inadequate public funding of poison control centers (PCCs) is almost as old as the facilities themselves. In the United States (US), the number of PCCs dropped from 661 to 55 (1978–2021) [1, 2] while in Germany only 7 [3] of the former 28 centers [4] still exist. The main reasons for reducing the number of PCCs were quality assurance reforms and insufficient public funding [5, 6]. Further reductions in the number of PCCs will likely affect the health care system, public budgets, and safety as their health and economic benefits have been shown: they can help to ensure a prompt and effective treatment [7], can save lives, help to reduce the unnecessary use of health care resources [8], lead to lower hospitalization rates [9] as well as reduced hospital length of stay [10, 11] and contribute to significant cost savings for the society [12–18]. Most of the studies regarding the economic benefits of PCCs are focused on the US health care system. The literature lacks support on how transferable these results are to the German or European context due to differences in the various countries' health care costs and health care structure. Studies from Europe are scarce, and for Germany only *Bindl *et al*.* [19] investigated the economic effects of a poison control center on health care costs in 1997. Deeper analysis and more recent data are needed for evidence-based decisions regarding the future funding of German PCCs. Therefore, this study aims to evaluate the cost–benefit ratio of a regional German PCC based on the current state of knowledge. We hypothesized that a scenario utilizing PCC consultation compared to a hypothetical scenario without PCC consultation would be associated with cost savings for the health care system, as unnecessary and more expensive health care contacts can be avoided.

## Methods

### Decision analysis

**Fig. 1:**
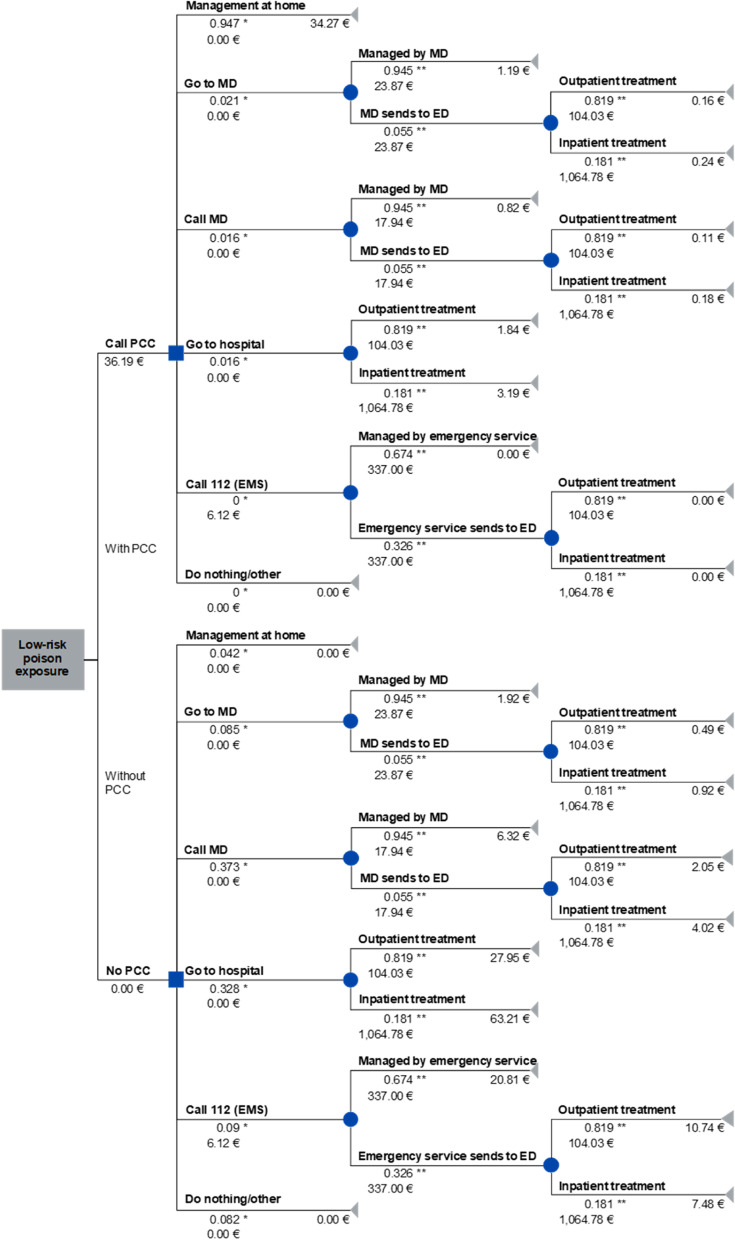
Decision tree with base case input values for low-risk poison exposures of laypersons in a situation utilizing PCC consultation and a hypothetical situation without PCC consultation. *Value derived from survey results; **Value derived from Berlin poison control center's data (2019); *ED* Emergency Department, *EMS* Emergency Medical Services, *MD* Medical Doctor

A decision tree model consistent with the simplified treatment pathways for exposures of laypersons (i.e., general public, no institutional callers) with poisoning concern was constructed (Fig. 1). The objective was to compare the costs of operating the PCC with its monetary benefit. For this, the perspective of the German health care system, including public health expenditures as well as the costs of private and statutory health insurance companies was taken. Analogous to comparable studies, the benefit was defined as the costs that can be avoided through the existence of the PCC. In the decision analysis, a situation utilizing PCC consultation and without PCC consultation was compared by calculating the average weighted financial outcome of each scenario. All input parameters used for the analysis can be found in the tables of Additional files 1 and 2. Calculations were performed using Microsoft Excel 2016 (Microsoft Corporation, Redmond, Washington, USA). The conducted analysis was based on the following assumptions:

1. All persons who call a medical doctor cause costs for medical advice. If the case is impossible to be solved by telephone, the medical doctor will send the affected person to the closest emergency department. Callers are not asked to come to the doctor's office for treatment.
2. If a layperson has already called the PCC, the attending medical doctor will not call the PCC again for this case.
3. Transition probabilities that could not be derived from the survey are assumed to be identical for the scenario with and without PCC consultation.

### Determination of transition probabilities

The data for calculating transition probabilities were collected through a survey of former callers at the Berlin PCC (Charité, Berlin). The Berlin PCC is handling about 45,000 human poison exposure cases per year. About half of these inquiries are made by laypersons. Most lay inquiries concern children aged ≤ 12 years (approximately 78%). An age definition ≤ 12 years for children is also used by the "Einheitlicher Bewertungsmaßstab (EBM)", a nationwide catalog on which the reimbursement of services provided by “National Association of Statutory Health Insurance Physicians (NASHIP)” accredited doctors in Germany is based on. Since most lay calls involve low-risk poison exposures, home management (i.e., self-monitoring at home) can be recommended in approximately 90% of the cases. The remaining cases are moderate to severe poisonings that require urgent medical help.

#### Interview of Berlin poison control center's callers

Since the COVID-19 pandemic reached Germany in early 2020, there has been a distinct change in the use of public health facilities [20]. Due to this fact, only data from before the COVID-19 outbreak in Germany were used for the study. The sample was limited to calls between December 2019 and January 2020 to ensure that respondents would remember their last call to the Berlin PCC. For the survey, only calls from laypersons regarding poisoning exposures of humans were included in which management at home was recommended, as this group reflects most of the laypersons calling the PCC.

For ethical reasons, cases with suicidal or criminal intent were not considered. Consequently, 2,419 cases were potentially eligible for the survey. Using the “KNIME Analytics Platform 4.2.2” (KNIME AG, Zurich, Switzerland) [21] 1,500 cases were randomly selected. A standardized questionnaire for the telephone survey was designed by the authors, which was pretested with 30 persons for content and comprehensibility by one of the authors. The same person, who performed the pretest, conducted a retrospective telephone survey for a five-week period between November 9 and December 15, 2020 subsequently. The aim was to acquire a minimum of 377 survey respondents, including the 30 pretested persons, to obtain a sufficiently large sample size. The sample size was calculated based on a total population of 19,378 calls in 2019 that met the criteria described previously (confidence level: 95%, margin of error: 5%). All potential survey participants were called up to three times at different times of the day.

After explaining the purpose of the survey and obtaining informed consent, the participants were asked: 1) Which medical service/s he/she had used before or after calling the PCC, 2) What he/she would have done if the PCC had not been available, 3) Whether the affected person was covered by private or statutory health insurance at the time of the call.

If a survey participant could not answer one of the open questions, the possible response options were read to the interviewed person. All responses were recorded in a standardized manner so that a systematic evaluation was possible after completing the survey. All persons who did not consent or were unable to participate (e.g., could not remember the former PCC call) were excluded from the survey.

During the evaluation, the response options "Call physician," "Call hospital," and "Call 116,117^1^" were merged in the main category "Call a medical doctor (MD)" to simplify the analysis. Moreover, the response options "Do research on the internet," "Call family/friends," "Do nothing/other," "Call a pharmacy," "Read the instructions on the packaging," and "I do not know" were combined in the main category "Do nothing/other" (Fig. 1 and Additional file 1).

#### Analysis of Berlin poison control center's data

Using the PCC's data (2019), the probabilities for the branches “Managed by MD/MD sends to ED”, “Outpatient treatment/Inpatient treatment” and “Managed by emergency service/Emergency service sends to ED” were determined, as they could not be obtained from the survey. To calculate the probabilities, the procedure recommended by the Berlin PCC for the different caller categories (doctors' office, hospital staff, and emergency services) were analyzed. Due to the fact that most lay inquiries have a low risk and concern children, only cases with asymptomatic patients aged ≤ 12 years were included to calculate transition probabilities (Fig. 1 and Additional file 1).

### Determination of costs and charges

All costs and charges used in the analysis are stated in Euro.

The variable and fixed costs per case were taken into account to calculate the PPC's average cost for one lay consultation. Based on a mixed calculation, staff costs of €14.79 were determined to process and follow-up a layperson's inquiry. Overhead costs (e.g., administrative and IT staff, rate for space rental and utilities, technical equipment, office supplies) amount to €21.40. Overall, this results in total costs of €36.19 per case.

By using the EBM catalog, charges for the treatment of persons with statutory health insurance were identified. Costs for privately insured persons were considered by multiplying corresponding EBM charges by a factor of 2.28 [23]. According to our own empirical data, most requests address children. For that reason, charges for the treatment of children (≤ 12 years) rather than for adults were used whenever possible. To determine appropriate charges according to the EBM catalog a pediatrician, a general practitioner, and a head of an emergency department for children was interviewed. Except for the branches "Call MD → Managed by MD" and "Call MD → MD sends to ED," no surcharges were included for the use of medical services outside of consultation hours and on weekends. Moreover, only direct costs were considered and indirect costs were excluded (e.g., driving and waiting time, increased length of stay in the hospital, work loss days). Charges for the actions “Call 112 (EMS)^2^” and “Call 112 (EMS) → Managed by emergency service/Emergency service sends to ED” were calculated based on data of the Berlin Fire Department [24]. The average costs for “Outpatient treatment” were derived from the literature [25], while the average costs for “Inpatient treatment” could be determined based on Charité controlling department data (2019). The actions “Management at home,” “Go to MD,” “Call MD,” “Go to hospital” and “Do nothing/other” were considered to be free of charge. Additional file 2 gives a detailed overview of all costs and charges included in the analysis.

### Sensitivity analysis

A sensitivity analysis based on the input parameters presented in the tables of Additional files 1 and 2 was conducted to test the robustness of study results to changes in cost drivers and probabilities. For that, IBM SPSS Statistics 27 (IBM Corporation, Armonk, New York, USA) and Microsoft Excel 2016 (Microsoft Corporation, Redmond, Washington, USA) was used. For cost drivers, a variation of  ± 25% was assumed. Based on the survey results and PCC's data (2019), the minimum and maximum values (= upper and lower bound of 95% confidence interval) for the transition probabilities were calculated using the bias-corrected and accelerated (BCa) bootstrap method with 1,000 re-samples. A deterministic one-way sensitivity analysis (DSA) for each input parameter (base case and min./max. values) with consideration to the recommended procedure of *Sendi and Clemen* [26] for chance nodes with more than two branches was performed. To be able to take 0%-probabilities into account, a min./max. range of 0% to 1% was estimated for them in the DSA. In addition to the DSA, a probabilistic sensitivity analysis (PSA) was conducted using a Monte Carlo simulation with 1,000 iterations and a normal distribution assumption.

## Results

**Fig. 2:**
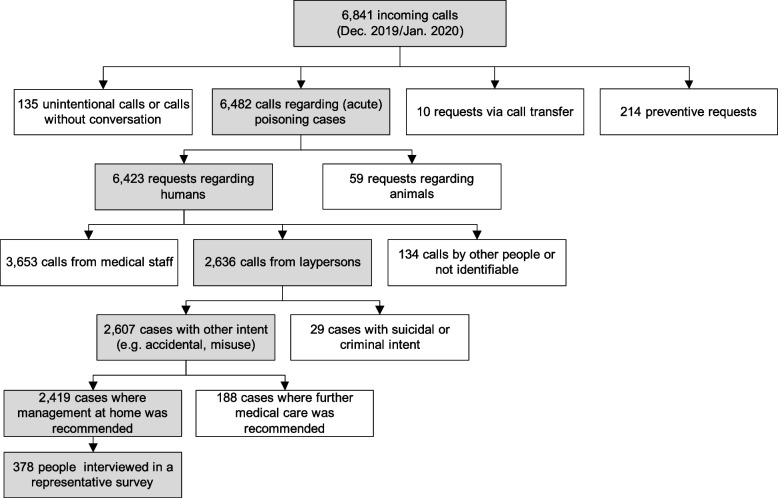
Participant flow chart of the telephone survey

From the randomly selected sample of 1,500 cases, 748 persons were contacted by telephone, of whom 310 could not be reached. Fifty persons were unable to answer (e.g., due to a lack of memory, false telephone number) and 10 persons did not gave their consent for participation. Finally, 378 persons participated fully in the survey. Figure 2 depicts the flow chart of survey participants. The participation rate of those reached by telephone was 86.3%.

Of the survey participants, 27 (7.1%) reported having sought other medical help before contacting the PCC and 358 (94.7%) stated that they did not use other medical services after PCC consultation. Although only low-risk poison exposure cases with the recommended procedure "Management at home" were included in the survey, 20 individuals (5.3%) used further medical help after calling the PCC (Table 1).

**Table 1:** Actual used medical services before or after consulting the Berlin PCC and alternative actions of callers in a hypothetical scenario of PCC's absence

	**Total number**	**Percentage**^a^
Action before calling the PCC		
Go to hospital	1	0.3%
Call MD	16	4.2%
Call 112 (EMS)	1	0.3%
Go to MD	2	0.5%
Management at home	-	0.0%
Use other medical service	7	1.9%
Do nothing/other	351	92.9%
∑	378	100.0%
Action after calling the PCC		
Go to hospital	6	1.6%
Call MD	6	1.6%
Call 112 (EMS)	-	0.0%
Go to MD	8	2.1%
Management at home	358	94.7%
Do nothing/other	-	0.0%
∑	378	100%
Alternative action without PCC		
Go to hospital	124	32.8%
Call MD	141	37.3%
Call 112 (EMS)	34	9.0%
Go to MD	32	8.5%
Management at home	16	4.2%
Do nothing/other	31	8.2%
∑	378	100%

*EMS* Emergency Medical Services, *MD* Medical Doctor

^a^ Values were rounded to one decimal place

**Fig. 3:**
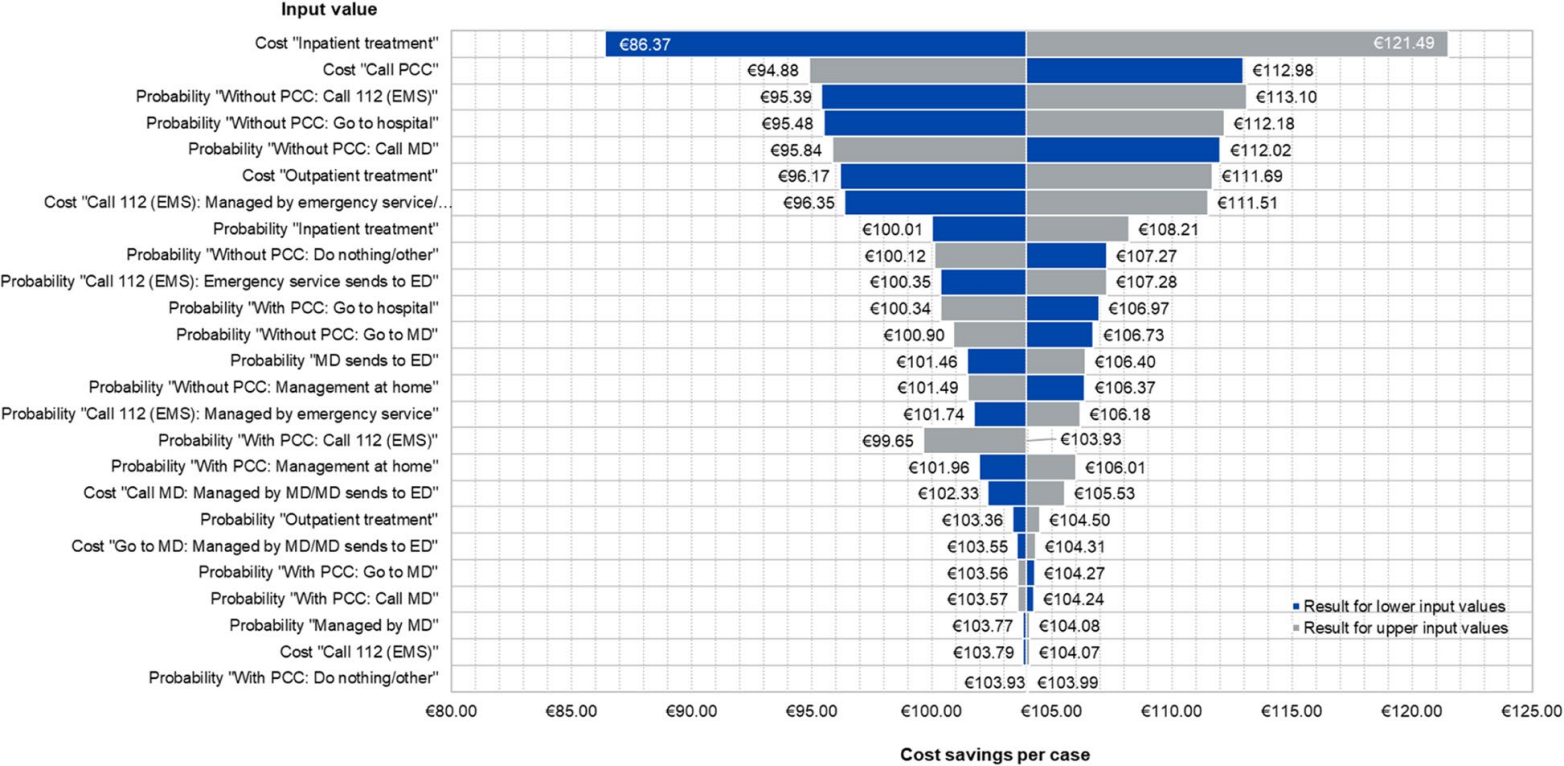
Results of deterministic sensitivity analysis: effect of lower/upper input-values on saved costs per case compared to a situation without PCC [reference point are saved costs of €103.93 for the base case scenario]. *ED* Emergency Department, *EMS* Emergency Medical Services, *MD* Medical Doctor, *PCC* Poison Control Center

In a hypothetical scenario without PCC, the majority would have chosen the alternative "Call MD" (37.3%), or "Go to hospital" (32.8%), followed by "Call 112 (EMS)" (9.0%), "Go to MD" (8.5%), "Do nothing/other" (8.2%) and "Management at home" (4.2%) (Table 1). Regarding the type of insurance, 327 persons (86.5%) indicated that the affected person had statutory health insurance at the time of the call, 50 survey participants (13.2%) had private health insurance, and one person (0.3%) did not want to provide information on the type of insurance. The decision tree analysis (Fig. 1) resulted in average costs for treating low-risk poison exposures of €41.99 for the scenario utilizing PCC consultation and €145.92 in the absence of PCC consultation (base case scenario). Including the services used after consulting the PCC, the cost-saving amounts to €103.93 per case. The cost–benefit ratio for the base case is 3.48 (€145.92/€41.99). The deterministic one-way sensitivity analysis showed that the costs of “Inpatient treatment” and “Call PCC” as well as the probability of “Without PCC → Call 112 (EMS)” have the most noticeable influence on the calculated cost difference compared to the base case (Fig. 3).

Overall, varying the input parameters by using the respective minimum and maximum values (Additional files 1 and 2) did not change the study findings to a relevant extent.

**Fig. 4:**
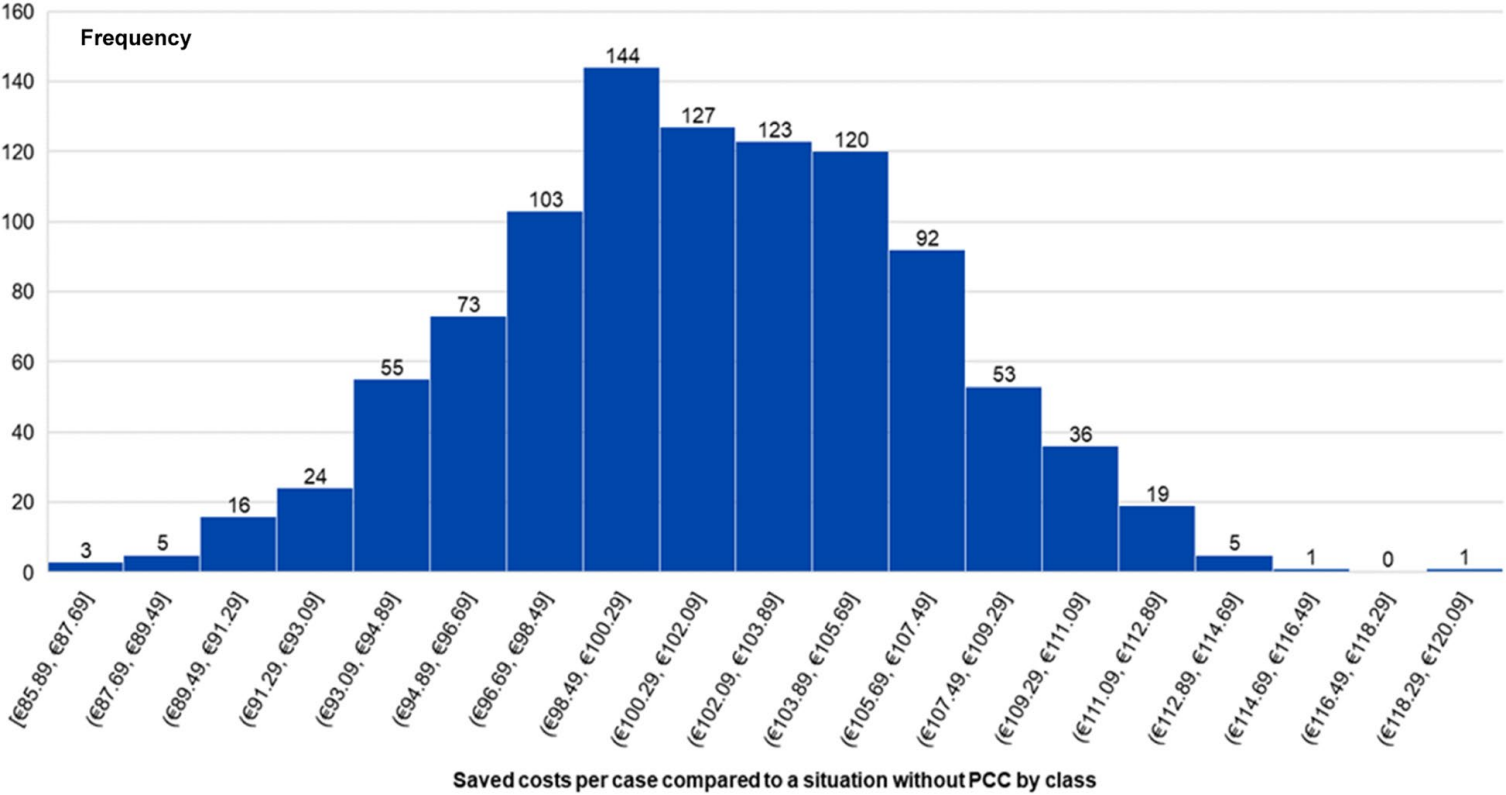
Results of probabilistic sensitivity analysis: saved costs per case compared to a situation without PCC and frequencies under randomly and simultaneously varying input-values. *PCC* Poison Control Center

Figure 4 illustrates the results of the probabilistic sensitivity analysis. The histogram depicts the result of the Monte Carlo simulation. The figure shows the frequencies of saved costs per case subdivided by class. An average cost saving of €101.45 per case was calculated by means of the PSA. Analogous to the DSA, a situation utilizing PCC consultation leads to relevant cost savings in all cases. Both sensitivity analyses give strong support for the robustness of the study results.

## Discussion

The present study aimed to perform an in-depth cost–benefit analysis for a German PCC (Charité, Berlin) based on current data and wanted to investigate how a regional PCC helps to relieve the health care system as well as reduces costs for the treatment of low-risk poison exposures. During our study, a comprehensive decision tree model was created, that can be used as a framework for future research, resulting in more accurate results than previous studies. Our survey revealed that laypersons with suspected poisoning would consult a health care professional in 87.6% of the cases if a PCC would not be available. This proportion is consistent with the results of comparable studies by *Descamps *et al*.* [16] from Belgium and *Toverud *et al*.* [7] from Norway, which found percentages of 86.2% and 85.3%, respectively. *Bindl *et al. [19] determined in 1997 that 96% of callers would have sought other medical services in the absence of the PCC. In recent studies, the higher percentage of persons who would have looked for help elsewhere can be attributed to the increased use of the Internet and the improved networking among people. Overall, our findings provide evidence that PCCs absence would lead to increased use of regular medical services, putting more pressure on the already overburdened emergency services [27].

It gives cause for concern that 4.2% of the surveyed persons would have treated the affected person themselves in a scenario without PCC. It can be assumed that this could not only lead to incorrect care for poison exposure cases, but could also cause complications or long-term damages. Accordingly, PCCs contribute to minimize medical costs in acute poisoning cases and reduce subsequent expenses caused by no or inadequate treatment.

Regarding insurance status, it was found that 86.5% of the affected persons were covered by statutory health insurance and 13.2% by private insurance at the time of the PCC call. These proportions correspond to the national average of 87.8% and 10.5% [28].

Taking into account the characteristics of our survey group, we calculated a cost–benefit ratio of 3.48. In comparable studies from the US, values ranged from 2.03 to 36 [12]. For European PCCs cost–benefit ratios from 0.76 to 5.70 were reported [7, 16, 19, 29]. Within the European studies, our result is closest to *Bindl *et al*.* [19], who found a ratio between 1.38 and 2.17 (calculation by authors based on study data). The differences in results are mainly due to the studies' differing methodological approaches and the variation in estimated costs. As proven by our sensitivity analysis, the costs of inpatient treatment, costs per PCC call, and the probability of calling 112 (EMS) in a situation without PCC have the most relevant influence on the calculated ratio.

### Limitations

A limitation of the study is that the modeled decision tree is a simplified representation of reality and only contains the most relevant elements for the cost–benefit analysis. Moreover, not all decision possibilities could be covered by empirical data. The option that the PCC is contacted multiple times for a specific case was not quantifiable and consequently could also not be considered. However, experience shows that medical professionals often call the PCC for cases on which laypersons already received advice. These additional calls of medical professionals can often avoid unnecessary emergency room visits and hospitalizations. Thus, it can be hypothesized that the calculated benefits of PCC would further increase if the study would take this fact also into account.

Our study only considered low-risk poison exposures from calling laypersons, leading to a one-sided view on the cost–benefit ratio of a PCC. Future work should therefore include moderate and severe poisoning cases, as well as calls from medical professionals in the cost–benefit analysis. The effect of a German PCC on outcomes such as hospital length of stay, patient mortality and morbidity, health-related quality of life and the number of poison exposures should additionally be investigated in further studies.

During the interview, it turned out that some persons had initially consulted other medical services before they were referred to the PCC. This circumstance could reduce the reported benefit. However, it can be assumed that prior use of other medical services could be nearly eliminated if awareness of PCC's emergency number would increase. For this reason, we have decided against the correction of the calculated benefit in this regard.

A further limitation of the study is that charges could not be transferred into costs. Consequently, costs and charges had to be mixed in the analysis.

Furthermore, only the direct costs from the health care system's perspective were used for calculating the benefit. Hence, the benefit estimates in the present study are rather conservative. A whole-society view incorporating indirect costs (e.g., waiting time, work loss days, perceived stress) and PCC's secondary benefits (e.g., through prevention work, toxicovigilance and health reporting) would lead to a more realistic cost–benefit ratio [30].

With regard to the study design, it is important to note that the time of day when a poisoning case occurs may influence the affected person's response and following treatment procedures. Therefore, the survey participants were selected randomly to minimize bias in the study results. An additional limitation of the study could result from a recall bias of the survey participants. To counteract this, all individuals who indicated that they could not accurately recall their contact with the PCC were excluded from the survey. Nevertheless, bias in the results likely occurred because parents often experience a suspected poisoning of their child as an acute emergency. Almost a year after knowing the child was treated at home, parents probably underestimate the alternative actions in a hypothetical situation without PCC [31].

## Conclusion

This study shows that PCCs reduce the usage of existing, more expensive medical services and thus reduce health care costs to a relevant extent. According to this finding, PCCs should be considered as an important supporting structure of the German health care system.

## Supplementary Information

**Additional file 1. Supplementary Table S1.** Transition probabilities of the decision tree.

**Additional file 2. Supplementary Table S2.** Cost/charges per case of the decision tree.

## Data Availability

The datasets generated during the current study are available from the corresponding author on reasonable request.

## Abbreviations

BCa: Bias-corrected and accelerated
DSA: Deterministic Sensitivity Analysis
EBM: Einheitlicher Bewertungsmaßstab
ED: Emergency Department
EMS: Emergency Medical Services
MD: Medical Doctor
PCC: Poison Conrol Center
PSA: Probabilistic Sensitivity Analysis

## Footnotes

1 116,117 is a nationwide telephone number for on-call outpatient medical services in Germany. The hotline connects local medical doctors with patients in urgent medical cases outside regular office hours (at night, on weekends, and public holidays) [22].

2 112 is the telephone number of emergency medical services (EMS) in Europe.
