# Supplementary material for: Advice for lay callers with low-risk poison exposures by a regional poison control center: the impact on health care expenditures
Source: Arch Public Health. 2022 Nov 30;80:243. doi: 10.1186/s13690-022-00994-0 (PMC9713099; doi:10.1186/s13690-022-00994-0)
Supplement: Supplementary file 1 — Additional file 1. Supplementary Table S1. Transition probabilities of the decision tree. [file 13690_2022_994_MOESM1_ESM.docx]

**Supplementary Online Content**

**Advice for lay callers with low-risk poison exposures by a regional poison control center: The impact on health care expenditures**

This supplementary material has been provided by the authors to give readers additional information about their work.

**Additional file 1: Supplementary Table S1** Transition probabilities of the decision tree

| Model item | Mean  Probability | 95 % CI  min./max.^a^ | Underlying calculations/ assumptions, sources |
| --- | --- | --- | --- |
| With PCC |  |  |  |
| Management at home | 94.7% | 92.9%, 96.6% | Calculated from survey results. |
| Go to MD | 2.1% | 1.1%, 3.2% | Calculated from survey results. |
| Go to MD → Managed by MD | 94.5% | 92.1%, 96.8% | Derived from poison control center's data (2019); Evaluation of the recommended procedure regarding calls from medical doctor's offices (asymptomatic patients ≤ 12 years). |
| Go to MD → MD sends to ED | 5.5% | 3.5%, 7.5% | Derived from poison control center's data (2019); Evaluation of the recommended procedure regarding calls from medical doctor's offices (asymptomatic patients ≤ 12 years). |
| Go to MD → MD sends to ED → Outpatient treatment | 81.9% | 80.7%, 83.1% | Derived from poison control center's data (2019); Evaluation of the recommended procedure regarding calls from hospital staff (asymptomatic patients≤ 12 years). |
| Go to MD → MD sends to ED → Inpatient treatment | 18.1% | 17.0%, 19.3% | Derived from poison control center's data (2019); Evaluation of the recommended procedure regarding calls from hospital staff (asymptomatic patients ≤ 12 years). |
| Call MD | 1.6% | 0.5%, 2.9% | Calculated from survey results. |
| Call MD → Managed by MD | 94.5% | 92.1%, 96.8% | Derived from poison control center's data (2019); Evaluation of the recommended procedure regarding calls from medical doctor's offices (asymptomatic patients ≤ 12 years). |
| Call MD → MD sends to ED | 5.5% | 3.5%, 7.5% | Derived from poison control center's data (2019); Evaluation of the recommended procedure regarding calls from medical doctor's offices (asymptomatic patients ≤ 12 years). |
| Call MD → MD sends to ED → Outpatient treatment | 81.9% | 80.7%, 83.1% | Derived from poison control center's data (2019); Evaluation of the recommended procedure regarding calls from hospital staff (asymptomatic patients ≤ 12 years). |
| Call MD → MD sends to ED →  Inpatient treatment | 18.1% | 17.0%, 19.3% | Derived from poison control center's data (2019); Evaluation of the recommended procedure regarding calls from hospital staff (asymptomatic patients ≤ 12 years). |
| Go to hospital | 1.6% | 0.5%, 2.9% | Calculated from survey results. |
| Go to hospital → Outpatient treatment | 81.9% | 80.7%, 83.1% | Derived from poison control center's data (2019); Evaluation of the recommended procedure regarding calls from hospital staff (asymptomatic patients ≤ 12 years). |
| Go to hospital → Inpatient treatment | 18.1% | 17.0%, 19.3% | Derived from poison control center's data (2019); Evaluation of the recommended procedure regarding |
|  |  |  | calls from hospital staff (asymptomatic patients ≤ 12 years). |
| Call 112 (EMS) | 0.0% | 0.0%, 0.1% | Calculated from survey results. The upper value (max.) of 0.1% was assumed for sensitivity analysis. |
| Call 112 (EMS) → Managed by  emergency service | 67.4% | 60.3%, 74.7% | Derived from poison control center's data (2019); Evaluation of the recommended procedure regarding calls from emergency services (asymptomatic patients ≤ 12 years). |
| Call 112 (EMS) → Emergency service  sends to ED | 32.6% | 26.2%, 38.6% | Derived from poison control center's data (2019); Evaluation of the recommended procedure regarding calls from emergency services (asymptomatic patients ≤ 12 years) |
| Call 112 (EMS) → Emergency service  sends to ED →  Outpatient treatment | 81.9% | 80.7%, 83.1% | Derived from poison control center's data (2019); Evaluation of the recommended procedure regarding calls from hospital staff (asymptomatic patients ≤ 12 years). |
| Call 112 (EMS) →  Emergency service  sends to ED →  Inpatient treatment | 18.1% | 17.0%, 19.3% | Derived from poison control center's data (2019); Evaluation of the recommended procedure regarding calls from hospital staff (asymptomatic patients ≤ 12 years). |
| Do nothing/other | 0.0% | 0.0%, 0.1% | Calculated from survey results. The upper value (max.) of 0.1% was assumed for sensitivity analysis. |
| Without PCC |  |  |  |
| Management at home | 4.2% | 2.6%, 5.8% | Calculated from survey results. |
| Go to MD | 8.5% | 6.1%, 11.1% | Calculated from survey results. |
| Go to MD → Managed by MD | 94.5% | 92.1%, 96.8% | Derived from poison control center's data (2019); Evaluation of the recommended procedure regarding calls from medical doctor's offices (asymptomatic patients ≤ 12 years). |
| Go to MD → MD sends to ED | 5.5% | 3.5%, 7.5% | Derived from poison control center's data (2019); Evaluation of the recommended procedure regarding calls from medical doctor's offices (asymptomatic patients ≤ 12 years). |
| Go to MD → MD sends to ED → Outpatient treatment | 81.9% | 80.7%, 83.1% | Derived from poison control center's data (2019); Evaluation of the recommended procedure regarding calls from hospital staff (asymptomatic patients ≤ 12 years). |
| Go to MD → MD sends to ED → Inpatient treatment | 18.1% | 17.0%, 19.3% | Derived from poison control center's data (2019); Evaluation of the recommended procedure regarding calls from hospital staff (asymptomatic patients ≤ 12 years). |
| Call MD | 37.3% | 32.8%, 41.8% | Calculated from survey results. |
| Call MD → Managed by MD | 94.5% | 92.1%, 96.8% | Derived from poison control center's data (2019); Evaluation of the recommended procedure regarding calls from medical doctor's offices (asymptomatic patients ≤ 12 years). |
| Call MD → MD sends to ED | 5.5% | 3.5%, 7.5% | Derived from poison control center's data (2019); Evaluation of the recommended procedure regarding calls from medical doctor's offices (asymptomatic patients ≤ 12 years). |
| Call MD → MD sends to ED → Outpatient treatment | 81.9% | 80.7%, 83.1% | Derived from poison control center's data (2019); Evaluation of the recommended procedure regarding calls from hospital staff (asymptomatic patients ≤ 12 years). |
| Call MD → MD sends to ED →  Inpatient treatment | 18.1% | 17.0%, 19.3% | Derived from poison control center's data (2019); Evaluation of the recommended procedure regarding calls from hospital staff (asymptomatic patients ≤ 12 years). |
| Go to hospital | 32.8% | 28.5%, 37.0% | Derived from the survey results. |
| Go to hospital → Outpatient treatment | 81.9% | 80.7%, 83.1% | Derived from poison control center's data (2019); Evaluation of the recommended procedure regarding calls from hospital staff (asymptomatic patients ≤ 12 years). |
| Go to hospital → Inpatient treatment | 18.1% | 17.0%, 19.3% | Derived from poison control center's data (2019); Evaluation of the recommended procedure regarding calls from hospital staff (asymptomatic patients ≤ 12 years). |
| Call 112 (EMS) | 9.0% | 6.3%, 11.9% | Calculated from survey results. |
| Call 112 (EMS) → Managed by  emergency service | 67.4% | 60.3%, 74.7% | Derived from poison control center's data (2019); Evaluation of the recommended procedure regarding calls from emergency services (asymptomatic patients ≤ 12 years). |
| Call 112 (EMS) → Emergency service  sends to ED | 32.6% | 26.2%; 38.6% | Derived from poison control center's data (2019); Evaluation of the recommended procedure regarding calls from emergency services (asymptomatic patients ≤ 12 years). |
| Call 112 (EMS) → Emergency service  sends to ED →  Outpatient treatment | 81.9% | 80.7%, 83.1% | Derived from poison control center's data (2019); Evaluation of the recommended procedure regarding calls from hospital staff (asymptomatic patients ≤ 12 years). |
| Call 112 (EMS) →  Emergency service  sends to ED →  Inpatient treatment | 18.1% | 17.0%, 19.3% | Derived from poison control center's data (2019); Evaluation of the recommended procedure regarding calls from hospital staff (asymptomatic patients ≤ 12 years). |
| Do nothing/other | 8.2% | 6.1%, 10.6% | Calculated from survey results. |

*ED* Emergency Department, *EMS* Emergency Medical Services, *MD* Medical Doctor

^a^ Min./max. values (= upper and lower bound of 95% confidence interval (CI)) were calculated using the bias-corrected and accelerated bootstrap (BCa) method with 1,000 bootstrap sample
