# Supplementary material for: Advice for lay callers with low-risk poison exposures by a regional poison control center: the impact on health care expenditures
Source: Arch Public Health. 2022 Nov 30;80:243. doi: 10.1186/s13690-022-00994-0 (PMC9713099; doi:10.1186/s13690-022-00994-0)
Supplement: Supplementary file 2 — Additional file 2. Supplementary Table S2. Cost/charges per case of the decision tree. [file 13690_2022_994_MOESM2_ESM.docx]

**Supplementary Online Content**

**Advice for lay callers with low-risk poison exposures by a regional poison control center: The impact on health care expenditures**

This supplementary material has been provided by the authors to give readers additional information about their work.

**Additional file 2: Supplementary Table S2** Cost/charges per case of the decision tree

| Model item | Costs/charges  per case | ± 25%  min./max | Underlying calculations/ assumptions, sources |
| --- | --- | --- | --- |
| Call PCC | €36.19 | €27.14, €45.24 | Own calculation based on operating costs of Charité PCC and staff costs per lay call (2019). |
| Management at home | €0.00 | €0.00, €0.00 | Management at home does not generate costs for the health system. |
| Go to MD | €0.00 | €0.00, €0.00 | The action of going to a medical doctor's office does not generate costs for the health system. |
| Go to MD → Managed by MD | €23.87 | €17.90, €29.84 | An expert interview with a pediatrician suggests that EBM code 04000 [[1](#_ENREF_1)] (2021) is usually billed for the treatment of children (≤ 4 years) or adolescents (5-18 years)  🡪 Mixed calculation:  (€25.03 + €15.80)/2 = €20.42 Calculation to account for the proportion of persons with statutory and private health insurance:  (0,868 * €20.42) + (0,132 * €20.42 * 2.28) = €23.87 |
| Go to MD → MD sends to ED | €23.87 | €17.90, €29.84 | If a medical doctor decides to send a patient to the ED, treatment charge is billed as well. 🡪 See calculations of “Go to MD → Managed by MD”. |
| Call MD | €0.00 | €0.00, €0.00 | The action of calling a medical doctor does not generate costs for the health system until the medical doctor takes care of the patient. |
| Call MD → Managed by MD | €17.94 | €13.46, €22.43 | Poison control center's data (2019) show that 70% of the calls are received in the time between 7 am and 7 pm (= regular office hours). An expert interview with a pediatrician suggests that EBM* code 01435 [[1](#_ENREF_1)] (2021) is billed for a telephone consultation during regular office hours. The authors assume that EBM codes 01100 and 01101 [[1](#_ENREF_1)] (2021) are billed at the other times 🡪 Weighted mixed calculation: (0.7 * €9.79) + (0.3 * (€21.80 € + €34.82)/2)) = €15.35  Calculation to account for the proportion of persons with statutory and private health insurance: (0,868 * €15.35) + (0,132 * €15.35 * 2.28) = €17.94 |
| Call MD → MD sends to ED | €17.94 | €13.46, €22.43 | If a medical doctor decides to send a patient to the ED, consultation charges are billed as well.  🡪 See calculations of “Call MD → Managed by MD”. |
| Go to hospital | €0.00 | €0.00, €0.00 | The action of going to a hospital does not generate costs for the health system until the patient is treated. |
| Outpatient treatment | €104.03 | €78.02, €130.04 | According to Haas et. al. (2015) [[2](#_ENREF_2)] the average outpatient treatment cost in the age group < 6 years is €89.00. Calculation to account for the proportion of persons with statutory and private health insurance:  (0,868 * €89.00) + (0,132 * €89.00 * 2.28) = €104.03 |
| Inpatient treatment | €1,064.78 | €798.59, €1,330.98 | Average costs of inpatients (≤ 12 years, principal diagnosis ICD-10-GM codes T36-T50 or T51-T65) treated at Charité in 2019 was €910.88. Calcu-lation to account for the proportion of persons with statutory and private health insurance: (0,868 * €910.88) + (0,132 * €910.88 * 2.28) = €1,064.78 |
| Call 112 (EMS) | €6.12 | €4.59, €7.65 | Own calculation based on costs of a cooperative police and fire control center [[3](#_ENREF_3)] (2020) and staff costs per call. According to an expert interview, the handling of a harmless poison exposure case takes on average 3 minutes and would be managed by an employee who costs €0.74 per minute [[4](#_ENREF_4)] (2019). Underlying assumptions: fire department bear half of the costs (straight-line depreciation over 25 year); 949.000 calls per year are received [[5](#_ENREF_5)] (2021). Calculation: (€3.700.000/949.000 calls) +  (3 min. * €0.74) = €6.12 |
| Call 112 (EMS) → Managed by  emergency service | €337.00 | €252.75 €, €421.25 | Own calculation based on charges (tariff units B 1.1, K 1.1 and K 1.2) of the Berlin fire department [[4](#_ENREF_4)] (2021) result in €337.00 per operation. |
| Call 112 (EMS) → Emergency service  sends to ED | €337.00 | €252.75, €421.25 | If the called emergency service decides to send a patient to the ED, charges are billed as well.  🡪 See calculations of “Call 112 (EMS) → Managed by emergency service”. |
| Do nothing/other | €0.00 | €0.00, €0.00 | “Do nothing/other” (e.g. call family/ friends, call a pharmacy, do research on the Internet) does not generate costs for the health system. |

*EBM* Einheitlicher Bewertungsmaßstab [[1](#_ENREF_1)], *ED* Emergency Department, *EMS* Emergency Medical Services, *MD* Medical Doctor, *PCC* Poison Control Center

**References**

1. KBV Kassenärztliche Bundesvereinigung. Einheitlicher Bewertungsmaßstab (EBM): Stand: 1. Quartal 2021. Berlin 2021.

2. Haas C, Larbig M, Schöpke T, Lübke-Naberhaus K-D, Schmidt C, Brachmann

M, et al. Gutachten zur ambulanten Notfallversorgung im Krankenhaus:

Fallkostenkalkulation und Strukturanalyse. Management Consult

Kestermann GmbH (MCK) in cooperation with Deutsche Gesellschaft

interdisziplinäre Notfall- und Akutmedizin e. V. (DGINA); 2015.

[https://www.dkgev.de/fileadmin/default/Mediapool/2_Themen/2.2_Finanzierung_und_Leistungskataloge/2.2.4._Ambulante_Verguetung/2.2.4.4._Ambulante_Notfallvehandlung_durch_Krankenhaeuser/2015-02-17_Gutachten_zur_ambulanten_Notfallversorgung_im
_Krankenhaus_2015.pdf](https://www.dkgev.de/fileadmin/default/Mediapool/2_Themen/2.2_Finanzierung_und_Leistungskataloge/2.2.4._Ambulante_Verguetung/2.2.4.4._Ambulante_Notfallvehandlung_durch_Krankenhaeuser/2015-02-17_Gutachten_zur_ambulanten_Notfallversorgung_im_Krankenhaus_2015.pdf). Accessed 16 Nov 2022.

3. Senatsverwaltung für Stadtentwicklung und Wohnen. Baumaßnahmen mit Gesamtkosten ab 100 Mio. Euro nach § 24 Abs. 3 LHO, 2955 D. 08.09.2020 ed. Berlin 2020. pp. 1-4.

4. Land Berlin. Gebührenordnung für die Benutzung von Einrichtungen

der Berliner Feuerwehr und die kostenersatzpflichtige Alarmierung/

Inanspruchnahme von Einrichtungen der Berliner Feuerwehr (Feuerwehrbenutzungsgebührenordnung - Fw BenGebO -) in der Fassung vom 13. April 1995; 2021. <https://gesetze.berlin.de/bsbe/document/jlr-FeuerwEBenGebOBEV11Anl-G1>. Accessed 16 Nov 2022.

1. Berliner Feuerwehr: Abteilung Einsatzsteuerung; 2021. <https://www.berliner-feuerwehr.de/ueber-uns/behoerdenstruktur/abteilung-einsatzsteuerung/>. Accessed 09 July 2021.
